# The Association Between Leukocyte Telomere Length and Cognitive Performance Among the American Elderly

**DOI:** 10.3389/fnagi.2020.527658

**Published:** 2020-10-30

**Authors:** Deng Linghui, Qiu Shi, Chen Chi, Liu Xiaolei, Zhou Lixing, Zuo Zhiliang, Dong Birong

**Affiliations:** ^1^National Clinical Research Center of Geriatrics, The Center of Gerontology and Geriatrics, West China Hospital, Sichuan University, Chengdu, China; ^2^Department of Urology, Institute of Urology and National Clinical Research Center for Geriatrics, West China Hospital, Sichuan University, Chengdu, China; ^3^Center of Biomedical Big Data, West China Hospital, Sichuan University, Chengdu, China; ^4^Department of Immunology and Microbiology, Guiyang College of Traditional Chinese Medicine, Guiyang, China

**Keywords:** aging, biomarker, telomere length, cognitive decline, elderly

## Abstract

**Background:**

Age-related cognitive decline begins in middle age and persists with age. Leukocyte telomere length (LTL) decreases with age and is enhanced by inflammation and oxidative stress. However, whether shorter LTL correlates with cognitive decline remains controversial.

**Aims:**

We aimed to investigate the relationship between LTL and cognitive decline in the American elderly.

**Methods:**

We used data from the 1999 to 2002 U.S. National Health and Nutrition Examination Survey (NHANES). We included participants aged 65–80 with available data on LTL and cognitive assessments. The cognitive function assessment used the digit symbol substitution test (DSST). We applied multivariate modeling to estimate the association between LTL and cognitive performance. Additionally, to ensure robust data analysis, we converted LTL into categorical variables through quartile and then calculated the *P* for trend.

**Results:**

After adjusting for age, cardiovascular disease (CAD) score, gender, race, body mass index (BMI), and educational level, LTL showed a positive correlation with DSST score (odds ratio [OR] 3.47 [0.14, 6.79], *P* = 0.04). Additionally, to further quantify the LTL–DSST interaction, we found a similar trend when LTL was regarded as a categorical variable (quartile) (*P* for trend = 0.03).

**Conclusion:**

LTL was associated with cognitive capabilities among the elderly, implying that LTL might be a biomarker of cognitive aging.

## Introduction

The aging global population presents a threat of increased disease and disability (Vos et al., 2012). Age-related cognitive decline begins in middle age and continues over time (Li et al., 2001), but studies have found individual differences in the severity of age-related cognitive decline under the influence of genetic and environmental factors (Foster, 2006; Connors et al., 2015; van der Wardt et al., 2015; Heywood et al., 2017). Studies have also suggested that oxidative stress and inflammation might influence aging and age-related memory disorders, which may explain why cognitive decline is highly associated with the elderly (Anstey et al., 2005; Peila and Launer, 2006; Lau et al., 2007; Rafnsson et al., 2007). However, the exact mechanism of cognitive decline remains unknown. Thus, to help reduce the risk of dementia and promote overall health among the elderly, it is essential to identify the potential predictive biomarkers of cognitive dysfunction.

Telomeres, which are repetitive nucleoprotein regions located at the ends of eukaryotic chromosomes, maintain genomic integrity and stability by protecting the end of the chromosome from illegitimate degradation and recombination (Jiang et al., 2018). Successive somatic cell divisions gradually abrase telomere length (TL) during aging (Miu et al., 2019). This cumulative age-interrelated TL shortening conduces to cellular senescence, an attribute of aging (López et al., 2013; Xu et al., 2018). Cellular lifespan is influenced by the length and stability of telomeres to some degree. Therefore, TL has been proposed as a candidate biomarker of aging (Xu et al., 2018). The TL shortening process was reported to accelerate under oxidative stress and inflammatory response and could serve as a record of the cumulative burden of oxidative stress along with inflammation (von Zglinicki et al., 2000; Aviv et al., 2006). Judging from longer telomeres protect cells from cellular senescence and death, it is reasonable to expect that they would also protect neuronal cells against oxidative stress and neurodegeneration that are related with cognitive decline (Collado et al., 2007). Research has reported the implication of shortened leukocyte TL (LTL) in multiple age-related diseases including neurodegenerative ones (Serrano and Andrés, 2004; Valdes et al., 2007; Shammas, 2011; Willeit et al., 2014).

Previous research on LTL and cognitive ability, predominantly in the elderly, has yielded inconsistent results. Studies have noticed a correlation between shortened LTL and age-related cognitive decline among the elderly (Valdes et al., 2010; Yaffe et al., 2011), but other studies have found such an association to be relatively small or absent (Mather et al., 2010; Harris et al., 2012). Therefore, we used the U.S. National Health and Nutrition Examination Survey (NHANES) database to investigate the relationship between LTL and cognitive function in a cohort of elderly individuals.

## Materials and Methods

### Data Source and Participant Selection

The NHANES is an ongoing cross-sectional survey of a nationally representative, non-institutionalized U.S. population conducted by the U.S. National Center for Health Statistics (NCHS). To promote overall representativeness, the NHANES uses a complex multi-stage probability sampling design. Its dataset combines five major factors: socio-demographic characteristics, physical examinations, dietary information, laboratory investigations, and interview or questionnaire data—with raw data that are processed in 2-year cycles and made publicly available online (Cawthon, 2002; Mazidi et al., 2017). The NHANES protocol was approved by the NCHS Research Ethics Review Board, and all participants provided written informed consent. More NHANES data and detailed information on survey methods are available on the center’s official website^1^.

In this study, we restricted our analysis to participants from 1999 to 2000 and from 2001 to 2002 cycles of the NHANES survey because only these cycles contained TL and cognitive testing information. Eligibility inclusion criteria required participants to be between ages 60 and 85 at the time of the survey, with high school education or above, and to have available data on both LTL and cognitive function examination.

### Telomere Measurements

With standardized procedures, purified DNA was acquired from whole blood and stored at −80°C before the LTL assay. Using quantitative polymerase chain reaction, we measured LTL relative to standard–reference DNA (T/S ratio) (Cawthon, 2002; Lin et al., 2010; Needham et al., 2013). The LTL was calculated as the mean T/S ratio, which is an approximation of average TL across all the chromosome ends. More details regarding the LTL quantification procedure and analytical methods are on the NHANES website (see footnote 1).

### Cognitive Function Assessment

We evaluated cognitive function using the digit symbol substitution test (DSST), an executive function subtest of the Wechsler Adult Intelligence Scale, Third Edition (WAIS III) (Oberlin et al., 2013). In this test, participants were given a key that paired symbols and numbers. Then, they were given a train of numbers and had to draw symbols under the corresponding numbers using the substitution key in 120s. The DSST score represents the correct number of symbols drawn, with a maximum score of 133. It is considered a sensitive test for cognitive disorder because it could record the participants’ response speed, associative learning, continuous attention, visual spatial skills, and memory abilities (Oberlin et al., 2013).

### Covariates

Multivariate models contain variables that might confound the link between LTL and cognitive ability. Educational level was coded as a level 2 categorical variable (high school and above). Cardiovascular disease (CAD) was also a potential confounder as it could affect both LTL and cognitive impairment. To avoid multicollinearity, we generated a comprehensive CAD variable that aggregated multiple risk factors. One score was assigned to each of the three current risk factors: history of hypertension, coronary heart disease, or stroke. Additionally, two points were added for diabetes. Scores ranged from 0 to 5 (Golub et al., 2019).

### Statistical Analysis

Statistical analysis was performed following CDC analytical reporting guidelines for complex NHANES data analysis^2^. We considered masked variance and used the recommended weighting scheme. Demographic characteristics among different TL groups (quartile) were compared using chi-square test or weighted linear regression model. To assess whether LTL is correlated with cognitive decline, our statistical analysis included three main steps. First, we employed weighted univariate and multivariate linear regression models. Multivariate models included model I (only gender, race, and educational level were adjusted) and model II [gender, race, educational level, age, CAD score, and body mass index (BMI) were adjusted]. Second, to account for non-linearity of cognitive decline and LTL, we conducted smooth curve fitting (penalized spline method) and weighted generalized additive model (GAM). Third, we performed subgroup analyses using weighted stratified linear regression models. For the continuous variable, we first converted it to a categorical variable according to the clinical cut point and then performed an interaction test.

To ensure that the data analysis is robust, we converted the TL into a categorical variable by quartile and calculated the P for trend. We did this to verify the TL results as a continuous variable and to observe the possibility of non-linearity.

All analyses were performed using the statistical software packages R^3^ (The R Foundation) and EmpowerStats^4^ (X&Y Solutions, Inc., Boston, MA, United States). *P*-values less than 0.05 (two-sided) were considered statistically significant.

## Results

### Participants’ Baseline Characteristics

Table 1 shows the weighted distribution of socio-demographic characteristics and other covariates of the selected participants in the NHANES 1999–2002 population.

**TABLE 1:** Baseline characteristics of participants.

Telomere length (T/S ratio)	Total sample	Q1 (0.389–0.759)	Q2 (0.759–0.880)	Q3 (0.880–1.026)	Q4 (1.026–3.001)	*P*-value
*N*	2,006	866	558	370	212	
Age [mean (SD)]	70.99 (7.54)	73.17 (7.96)	71.09 (7.71)	69.76 (7.53)	68.81 (7.30)	<0.001
BMI	28.78 (6.09)	28.11 (5.15)	28.12 (5.69)	28.29 (5.50)	28.67 (5.70)	0.585
Gender						<0.001
Male	6,614(48.88%)	470(54.27%)	289(51.79%)	156(42.16%)	84(39.62%)	
Female	6,916(51.12%)	396(45.73%)	269(48.21%)	214(57.84%)	128(60.38%)	
Educational level						0.447
High school	6,540(48.34%)	471(54.39%)	280(50.18%)	200(54.05%)	114(53.77%)	
Above high school	6,990(51.66%)	395(45.61%)	278(49.82%)	170(45.95%)	98(46.23%)	
CAD score [mean (SD)]	1.20 (1.18)	1.01 (1.10)	0.98 (1.09)	0.91 (0.99)	1.03 (1.12)	0.476
Diabetes						0.238
No	10,281(78.53%)	693(82.60%)	455(84.10%)	314(87.22%)	172(82.69%)	
Yes	2,810(21.47%)	146(17.40%)	86(15.90%)	46(12.78%)	36(17.31%)	
Hypertension						0.509
No	5,495(40.72%)	426(49.31%)	276(49.64%)	178(48.37%)	93(43.87%)	
Yes	7,998(59.28%)	438(50.69%)	280(50.36%)	190(51.63%)	119(56.13%)	
Hyperlipidemia						0.955
No	12,012(89.68%)	762(89.96%)	496(89.69%)	327(89.59%)	191(90.95%)	
Yes	1,383(10.32%)	85(10.04%)	57(10.31%)	38(10.41%)	19(9.05%)	
Stroke						0.556
No	12,349(91.49%)	801(92.60%)	518(93.33%)	349(94.32%)	201(94.81%)	
Yes	1,148(8.51%)	64(7.40%)	37(6.67%)	21(5.68%)	11(5.19%)	
Race						0.002
Mexican American	1,248(9.22%)	101(11.66%)	64(11.47%)	48(12.97%)	14(6.60%)	
Other Hispanic	858(6.34%)	28(3.23%)	13(2.33%)	8(2.16%)	12(5.66%)	
Non-Hispanic White	7,918(58.52%)	603(69.63%)	373(66.85%)	255(68.92%)	131(61.79%)	
Non-Hispanic Black	2,717(20.08%)	117(13.51%)	90(16.13%)	53(14.32%)	52(24.53%)	
Other Race	789(5.83%)	17(1.96%)	18(3.23%)	6(1.62%)	3(1.42%)	

*CAD score: 1 score was appointed for each of the three current risk factors, including history of hypertension, coronary heart disease, or stroke. Additionally, two points were added for diabetes. Scores range from 0 to 5.*

The participants in this sample averaged 70.99 ± 7.54 years old, with women representing 51.1%. Among different TL groups (quartile, Q1–Q4), the following distributions were similar: educational level, BMI, CAD score, history of hypertension, diabetes, hyperlipidemia, and stroke. Compared with groups Q1 and Q2, groups Q3 and Q4 were younger and had a higher percentage of females. Regarding cognitive performance, DSST scores ranged from 0 to 117, with a mean of 44.56 (SD = 17.68). In addition, the maximum and minimum T/S ratio values were 3.00 and 0.39, respectively, with a mean of 0.91 (SD = 0.22).

### Univariate Linear Regression Analysis

We first assessed a univariate linear regression model to evaluate the relationship between LTL and DSST. The results found that female gender, educational level, and LTL are positively correlated with DSST score, whereas male gender, age, and CAD score are negatively associated with DSST score (see Supplementary Table S1 in the Appendix).

### Multivariate Linear Regression Analysis of the LTL–DSST Relationship

Table 2 shows the coefficients of the association between LTL as continuous variable and DSST score. Our multivariate linear regression analysis showed a negative correlation between LTL and DSST score in the crude model (odds ratio [OR] 9.51 [5.82–13.19], *P* < 0.01) (see Table 2). In the adjusted model I (adjusted by gender, race, and educational level) and model II (adjusted by gender, race, educational level, age, CAD score, and BMI), the results remained stable (OR 10.27 [6.83, 13.72], *P* < 0.01; OR 3.47 [0.14, 6.79], *P* = 0.04).

**TABLE 2:** Multivariable linear regressions analysis of the LTL–DSST relationship.

Exposure	Non-adjusted model	Model I	Model II
Telomere length (T/S ratio)	9.51 (5.82, 13.19), <0.01	10.27 (6.83, 13.72), <0.01	3.47 (0.14, 6.79), 0.04
**Telomere length (T/S ratio)**			
Q1 (0.39–0.76)	Reference	Reference	Reference
Q2 (0.76–0.88)	2.93 (1.01, 4.85), <0.01	2.87 (1.08, 4.66), 0.01	0.88 (−0.81, 2.58), 0.31
Q3 (0.88–1.02)	4.52 (2.30, 6.73), <0.01	4.43 (2.37, 6.50), <0.01	1.01 (−0.96, 2.97), 0.32
Q4 (1.02–3.00)	4.75 (2.02, 7.49), <0.01	5.87 (3.32, 8.43), <0.01	2.39 (−0.04, 4.82), 0.05
*P* for trend	<0.001	<0.001	0.03

*Model I adjusted for gender, race, and educational level; model II adjusted for gender, race, educational level, age, CAD score, and BMI.*

Additionally, to further detect the correlation of LTL–DSST interaction, we stratified the participants into four groups by the 25th, 50th, and 75th LTLx percentiles (0.39–0.76, 0.76–0.88, 0.88–1.03, and 1.03–3.00, respectively). LTL was regarded as a categorical variable (quartile), and a similar trend was observed (*P* for trend = 0.03; see Table 2). On multivariable analysis, participants in quartile 4 had 139% higher odds of getting higher DSST score than those in quartile 1.

### Analyses of LTL–DSST Non-linear Relationship

It is essential to analyze non-linear relationships for continuous variables. A weighted GAM and smooth curve fitting (penalized spline method) were used to investigate the non-linear relationship between LTL and DSST scores. After adjusting for gender, age, educational level, race, CAD score, and BMI, we did not observe a non-linear relationship between LTL and DSST (see Supplementary Figure S1 in the Appendix).

### Subgroup Analyses

Table 3 shows our subgroup analysis results. We found that, after adjusting for potential confounders, the interaction test was not statistically significant for age, educational level, gender, race, CAD score, and BMI (*P* for interaction > 0.05). Thus, we do not detect any substantial evidence to prove that there are systematic differences in associations in different subgroups of the population, which means our main results are stable.

**TABLE 3:** Effect size of prespecified and exploratory subgroups.

Characteristic	No. of participants	Effect size (95% CI), *P*-value	*P* for interaction
Age			0.946
<70	781	5.26 (0.24, 10.28), **0.04**	
≥70	864	5.02 (0.38, 9.66), **0.03**	
Gender			0.815
Male	816	3.04 (−1.87, 7.95), 0.23	
Female	829	3.04 (−1.87, 7.95), 0.23	
Race			0.434
Mexican American	176	−0.13 (−11.76, 11.51), 0.98	
Other Hispanic	50	−9.90 (−25.39, 5.59), 0.21	
Non-Hispanic White	1,152	4.06 (0.17, 7.95), **0.04**	
Non-Hispanic Black	229	6.26 (−2.23, 14.75), 0.15	
Other race	38	5.85 (−26.79, 38.50), 0.73	
CAD score			0.583
<3	1,463	3.73 (0.18, 7.29), **0.04**	
≥3	182	1.01 (−8.16, 10.18), 0.83	
Educational level			0.206
High school	845	5.27 (0.92, 9.63), **0.02**	
Above high school	800	1.10 (−3.85, 6.06), 0.66	
BMI			0.480
Lower	439	1.80 (−4.03, 7.62), 0.55	
Higher	1,206	4.30 (0.32, 8.27), **0.03**	

*Gender, race, educational level, age, CAD score, and BMI were adjusted. Bold data mean *p* value < 0.5.*

## Discussion

This study’s central finding suggests that cognitive decline correlated significantly with shorter LTL among elderly individuals, and the findings were found to be robust after adjusting for various potential confounders.

Previous studies have noted the role of LTL in a series of age-related chronic diseases including neurodegenerative and CAD (Panossiana et al., 2003; Benetos et al., 2004; Martin et al., 2006; Brouilette et al., 2007; van der Harst et al., 2007; Fitzpatrick et al., 2011). Consistent with our results, some previous studies found that compared with healthy individuals, patients with cognitive disorder [including mild cognitive impairment (MCI) and Alzheimer’s disease (AD)] have truncated TLs in peripheral blood leukocytes (Honig et al., 2006; Grodstein et al., 2008; Mather et al., 2010; Valdes et al., 2010), mononuclear cell (Panossiana et al., 2003), and even in brain tissue (Thomas et al., 2008), while other studies did not observe a correlation between cognitive performance and LTL (Zekry et al., 2010; Hochstrasser et al., 2012; Movérare-Skrtic et al., 2012).

Furthermore, genetics might be a potential confounder in such a correlation in view of the heritable characteristics of LTL (Jeanclos et al., 2000; Nawrot et al., 2004). However, studies have also found significant differences in cognitive scores between twins with inconsistent TL, and the results confirmed that the correlation observed between decreased cognitive ability and shortened TL is robust to age and possible confounding factors.

The precise mechanism underlying the correlation between shorter LTL and cognitive decline remains unclear. Regarding TL, apart from the erosion of telomeres with cell division, telomeres are highly sensitive to damage by oxidative stress and inflammation (Saretzki and Von Zglinicki, 2002; Aviv et al., 2006). Oxidative stress enhances telomere attrition with each cell division (Saretzki and Von Zglinicki, 2002), whereas inflammation entails increased leukocyte turnover and subsequently heightens telomere attrition. An *in vitro* study reported that the telomere shortening process could be modulated by oxidative stress (Kawanishi and Oikawa, 2004), and many proinflammatory markers, such as interleukin (IL)-6, were negatively correlated with TL (O’Donovan et al., 2011). Evidence showed that short/dysfunctional telomeres in peripheral immune cells as well as in microglia could contribute to cellular senescence that are linked to higher secretion of proinflammatory mediators that play an important role in the etiopathogenesis and progression of cognitive impairment (Collado et al., 2007; Jurk et al., 2012; Weng, 2012; Boccardi et al., 2015). To some extent, LTL might hinge on the cumulative burden of inflammation and oxidative stress across the lifespan (Sultana et al., 2009). Therefore, oxidative stress and inflammation might be the potential thread linking TL shortening with cognitive dysfunction.

Several alternative pathways may underlie the pathogenesis of cognitive impairment. The hippocampus is closely associated with cognitive function and is regarded as a key area affected by aging. It has been reported that TL is independently associated with cortical epithelial marginal areas (including hippocampus and orbitofrontal cortex areas), which overlaps with brain regions related to cognitive impairment psychopathology. Previous animal research reported that telomerase-deficient mice models showed neuronal loss in the hippocampus and frontal cortex linked with short-term memory deficits (Rolyan et al., 2011), whereas telomerase reactivation could reverse aging-related cognitive deficits (Jaskelioff et al., 2011).

This study exhibits multiple strengths. The multi-ethnic, national representative data from NHANES allow our findings to be extrapolated to a broader population. LTL and cognitive assessment were measured in a large sample, offering high statistical power to explore their connection. Additionally, with the NHANES’s rigorous methodology and comprehensive quality procedures, we adjusted for several potential confounders by capitalizing on its abundant data.

However, this study also has several imitations. First, its primary constraint is its cross-sectional design, precluding inferences about causation. Second, LTL comes from one measurement rather than assessed longitudinally, which may provide key insights into the aging process. The shortening rate of LTL may be a more relevant indicator of the wear and tear that results in accelerated biological aging than TL measured at one point in time. Third, because TL maintenance or loss is related to many environmental factors, TL is also controlled by genetics and varies widely between individuals (Graakjaer et al., 2004). Therefore, ideally, one must consider all these intermediate parameters before attributing the differences in TL to a particular phenomenon. Fourth, a potential limitation is the use of only one specific measure of cognitive ability rather than multiple ones, and the type of cognitive measurement might lead to different findings. Accompanying tests, such as the Mini Mental Status Examination (MMSE), could help to assess cognitive function more comprehensively. However, DSST performance seems to be sensitive in reflecting attention/concentration, visual discrimination, information processing speed, and working memory and is especially suitable for screening MCI in the elderly (Salthouse, 1996). Moreover, it was reported to be less sensitive to educational level (Hoyer et al., 2004). Thus, the DSST scale may represent a relatively comprehensive cognitive scale (Launer et al., 2011). Future studies are required to determine the relationship between LTL and cognitive decline, which may reveal new knowledge on the effect of oxidative stress and inflammation on health and longevity.

## Conclusion

Using the NHANES database, this study found a significant association between LTL and cognitive performance in the elderly after adjusting for potential confounding factors. Further high-quality studies need to be conducted to better understand the pathophysiology of such a correlation.

## Data Availability Statement

Publicly available datasets were analyzed in this study. These data can be found here: https://wwwn.cdc.gov/nchs/nhanes/tutorials/default.aspx.

## Ethics Statement

The studies involving humans were reviewed and approved by the National Center for Health Statistics Research Ethics Review Board. Patients/participants provided their written informed consent to participate in the study.

## Author Contributions

DBR, DLH, and QS designed the study. DLH and QS drafted the manuscript. DLH, QS, and CC acquired the data. CC, DLH, and QS analyzed the data. DBR, CC, LXL, ZLX, and ZZL revised the manuscript critically for important intellectual content. All authors contributed to the article and approved the submitted version.

## Conflict of Interest

The authors declare that the research was conducted in the absence of any commercial or financial relationships that could be construed as a potential conflict of interest.

## Footnotes

1 http://www.cdc.gov/nchs/nhanes.htm

2 https://wwwn.cdc.gov/nchs/nhanes/tutorials/default.aspx

3 http://www.R-project.org

4 http://www.empowerstats.com
