# Supplementary material for: The Association Between Leukocyte Telomere Length and Cognitive Performance Among the American Elderly
Source: Front Aging Neurosci. 2020 Oct 30;12:527658. doi: 10.3389/fnagi.2020.527658 (PMC7661855; doi:10.3389/fnagi.2020.527658)

Appendix file

Table S1: Urivariable Linear Regressions Analysis

| Exposure | Univariate |
| --- | --- |
| Age | -0.74 (-0.82, -0.65), <0.0001 |
| CAD score | -3.31 (-3.99, -2.63), <0.0001 |
| Gender |  |
| male | Reference |
| female | 2.31 (0.86, 3.75), 0.0018 |
| Educational level |  |
| High school | Reference. |
| Above high school | 9.30 (7.90, 10.70), <0.0001 |
| Race |  |
| Mexican American | Reference. |
| Other Hispanic | -2.16 (-6.85, 2.53), 0.3662 |
| Non-Hispanic White | 6.28 (4.00, 8.56), <0.0001 |
| Non-Hispanic Black | -6.32 (-9.11, -3.52), <0.0001 |
| Other Race | 5.00 (0.16, 9.85), 0.0432 |
| BMI | 0.02 (-0.12, 0.16), 0.7859 |
| Telomere | 9.51 (5.82, 13.19), <0.0001 |

Figure S1 The non-linear relationship between LTL and DSST using the generalized additive model.


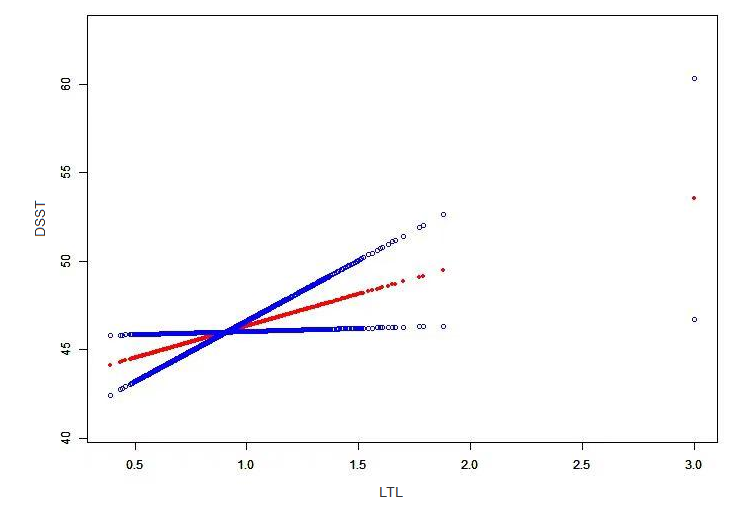

Supplement: Supplementary file 1 [file Table_1.DOCX]
